# GABAergic Inhibition of Presynaptic Ca^2+^ Transients in Respiratory PreBötzinger Neurons in Organotypic Slice Cultures

**DOI:** 10.1523/ENEURO.0154-21.2021

**Published:** 2021-08-21

**Authors:** Carlos Daniel Gómez, Camilla Mai Rasmussen, Jens C. Rekling

**Affiliations:** Department of Neuroscience, University of Copenhagen, Copenhagen N DK-2200, Denmark

**Keywords:** breathing, GABA_A_, GECI, jGCaMP7, presynaptic inhibition

## Abstract

GABAergic somatodendritic inhibition in the preBötzinger complex (preBötC), a medullary site for the generation of inspiratory rhythm, is involved in respiratory rhythmogenesis and patterning. Nevertheless, whether GABA acts distally on presynaptic terminals, evoking presynaptic inhibition is unknown. Here, we begin to address this problem by measuring presynaptic Ca^2+^ transients in preBötC neurons, under rhythmic and non-rhythmic conditions, with two variants of genetically encoded Ca^2+^ indicators (GECIs). Organotypic slice cultures from newborn mice, containing the preBötC, were drop-transduced with jGCaMP7s, or injected with jGCaMP7f-labeling commissural preBötC neurons. Then, Ca^2+^ imaging combined with whole-cell patch-clamp or field stimulation was obtained from inspiratory preBötC neurons. We found that rhythmically active neurons expressed synchronized Ca^2+^ transients in soma, proximal and distal dendritic regions, and punctate synapse-like structures. Expansion microscopy revealed morphologic characteristics of bona fide synaptic boutons of the en passant and terminal type. Under non-rhythmic conditions, we found that bath application of the GABA_A_ receptor agonist muscimol, and local microiontophoresis of GABA, reduced action potential (AP)-evoked and field stimulus-evoked Ca^2+^ transients in presynaptic terminals in inspiratory neurons and commissural neurons projecting to the contralateral preBötC. In addition, under rhythmic conditions, network rhythmic activity was suppressed by muscimol, while the GABA_A_ receptor antagonist bicuculline completely re-activated spontaneous activity. These observations demonstrate that the preBötC includes neurons that show GABAergic inhibition of presynaptic Ca^2+^ transients, and presynaptic inhibition may play a role in the network activity that underlies breathing.

## Significance Statement

Presynaptic inhibition mediated by ion channels or receptors in the presynaptic membrane serves to control the strength of synaptic transmission, transiently or long-term in many neural circuits. However, it is unclear whether central pattern generator (CPG) networks controlling rhythmic movements make use of modulation of presynaptic Ca^2+^. Here, we use genetically encoded Ca^2+^ sensors expressed in presynaptic terminals in organotypic slice cultures, to study the effect of GABA_A_ receptor agonists on inspiratory neurons in the breathing CPG in the brainstem preBötzinger complex (preBötC). Under non-rhythmic conditions, we show that muscimol and GABA reduce presynaptic Ca^2+^ evoked by action potentials (APs) and field stimulus in inspiratory neurons, suggesting that neurons in the breathing CPG may rely on presynaptic inhibition to control transmitter release.

## Introduction

Synaptic inhibition plays an important role in most rhythmic networks, including the brainstem network that generates and pattern respiratory rhythm (Abdala et al., 2015; Anderson and Ramirez, 2017; Ikeda et al., 2017; Del Negro et al., 2018). The ventrolateral medulla contains physiologically and neurochemically identified groups of respiratory neurons in the ventral respiratory column (VRC), which comprise subsets of GABAergic and glycinergic neurons (Smith et al., 2009; Morgado-Valle et al., 2010; Koizumi et al., 2013). An estimated 50% of inspiratory neurons in the preBötzinger complex (preBötC), an essential site for inspiratory rhythm generation, contains GABA or glycine (Kuwana et al., 2006; Winter et al., 2009; Morgado-Valle et al., 2010; Baertsch et al., 2018), and some may be co-transmitting GABA and glycine (Hirrlinger et al., 2019). Expiratory neurons in the adjacent Bötzinger complex use GABAergic (Ellenberger, 1999) and glycinergic (Ezure et al., 2003) transmission to inhibit spinal and medullary neurons involved in respiratory pattern generation (Ausborn et al., 2018) and active expiration (Flor et al., 2020). Pharmacological blocking and optogenetic experiments suggest that synaptic inhibition is not obligatory in the network generation of inspiratory rhythm (Shao and Feldman, 1997; Janczewski et al., 2013) but has phase-dependent effects on the synchronization of preBötC neurons (Ashhad and Feldman, 2020), respiratory frequency and amplitude (Sherman et al., 2015; Cregg et al., 2017; Baertsch et al., 2018). Several *in vivo* and *in vitro* experiments point to a crucial role of synaptic inhibition in generating the three-phase rhythm of eupnea (Richter and Smith, 2014; Marchenko et al., 2016), with phasic inhibition ensuring a stable rhythm generation, and integration of respiration with other ongoing motor programs. These effects of synaptic inhibition on respiratory rhythm generation and patterning are generally assumed to be because of somatodendritic inhibitory effects of GABAergic and glycinergic transmission (Liu et al., 2002). However, no studies have approached the question of whether GABA or glycine may affect presynaptic terminals directly, as is the case in presynaptic inhibition used in several other networks in the CNS, retina, and spinal cord (Kullmann et al., 2005). Here, we use organotypic slice cultures of the brainstem to investigate whether presynaptic GABAergic inhibition exists in inspiratory neurons in the preBötC, measuring the effect of GABA_A_ receptor ligands on presynaptic Ca^2+^ transients.

Genetically encoded Ca^2+^ indicators (GECIs) have improved the measurement of Ca^2+^ in functioning neurons under *in vitro* and *in vivo* conditions (Mollinedo-Gajate et al., 2019). In particular, recent GECI variants show enhanced labeling and signal-to-noise ratio in neuronal populations and microcompartments (Dana et al., 2019). Thus, jGCaMP7 variants with slow and fast kinetics (jGCaMP7s and jGCaMP7f, respectively), label small neuronal processes, including presynaptic boutons and dendritic spines, and have been employed to report on Ca^2+^ fluctuations in intact networks in the fly and mouse brain. The use of GECIs has also benefited from improved viral vectors that can deliver the genetic material to neurons with specificity (Chen et al., 2019). In particular, adeno-associated virus (AAV) vectors have proved advantageous in effectively transducing neurons and labeling projection neurons retrogradely using the AAV retrograde serotype (Tervo et al., 2016).

Here, we use two jGCaMP7 GECI variants expressed in organotypic slice cultures from mice containing the preBötC, via AAV9 and AAV retrograde transductions, to investigate the effect of GABA_A_ receptor ligands on presynaptic Ca^2+^ transients in inspiratory neurons. We show that, under non-rhythmic conditions, bath applied muscimol, a GABA_A_ receptor agonist, and microiontophoretically applied GABA, reduce action potential (AP)-evoked and field stimulus-evoked presynaptic Ca^2+^ in inspiratory preBötC neurons, including commissural neurons projecting to the contralateral preBötC. This effect on presynaptic Ca^2+^ demonstrates that synaptic inhibition in the breathing oscillator may involve GABAergic presynaptic inhibition.

## Materials and Methods

### Organotypic slice cultures

All animal procedures were performed in accordance with the University animal care committee’s regulations. Organotypic slice cultures containing the preBötC were prepared as previously described (Phillips et al., 2016, 2018). In brief, United States Naval Medical Research Institute (NMRI) mice postnatal ages P2.5–P10.5, of either sex, were anesthetized with isoflurane (Baxter), and immediately dissected in sterile-filtered chilled dissection artificial CSF (d-aCSF) containing the following: 135 mm glycerol, 2.5 mm KCl, 1.2 mm NaH_2_PO_4_, 30 mm NaHCO_3_, 5 mm HEPES acid, 15 mm HEPES base, 25 mm D-glucose, 5 mm sodium ascorbate, 2 mm thiourea, 3 mm sodium pyruvate, 10 mm MgSO_4_, and 0.5 mm CaCl_2_; pH 7.3, equilibrated by bubbling with 95% O_2_/5% CO_2_. A single transverse slice of the brainstem, 400 μm in thickness, was taken at the level of the preBötC using a vibrating microtome (ThermoFisher Scientific Microm 650V, RRID:SCR_008452). One to four transverse brainstem slices were placed onto semi-porous culture well inserts via the Stoppini interface method (Millipore catalog #PIC03050, RRID:SCR_008983), and maintained in sterile-filtered organotypic culture media containing: 95% Neurobasal-A medium (ThermoFisher Scientific, catalog #10888-022, RRID:SCR_008452), 2% B-27 supplement (ThermoFisher Scientific, catalog #17504044, RRID:SCR_008452), 2 mm GlutaMAX (ThermoFisher Scientific, catalog #35050-038, RRID:SCR_008452), 0.5 μm T3 (Sigma-Aldrich, catalog #T6397, RRID:SCR_008988), 0.5 μm T4 (Sigma-Aldrich, catalog #T1775, RRID:SCR_008988), 200 U/ml penicillin, 5 μg/ml streptomycin, and 10 mm HEPES and pH was set at 7.25. The cultures were treated with 10 μm MK-801 (Sigma-Aldrich, catalog #M107, RRID:SCR_008988) for the first 3 d in culture, and fresh culture media were supplied every 48 h thereafter until experimentation. The cultures were kept in a sterile, humidified incubator at 35°C, with 5% CO_2_.

### Labeling with organic Ca^2+^ dyes and AAV constructs

Loading solution for the membrane-permeable Ca^2+^ indicator Fluo-8, AM was prepared by combining 30 μl of a 10 mm stock solution containing Fluo-8, AM in DMSO (AAT Bioquest, catalog #21081) with 3.5-μl cremophore EL (Fluka, catalog #27963) and 7.5 μl of 20% pluronic acid in DMSO (AAT Bioquest, catalog #20052). These 41 μl of dye solution were then diluted in 1.5 ml of loading aCSF (l-aCSF) containing 10 μm MK-571 (a transporter blocker that improves Ca^2+^ indicator dye uptake, Sigma-Aldrich, catalog #M7571, RRID:SCR_008988). The l-aCSF consisted of the following: 129 mm NaCl, 3 mm KCl, 25 mm NaHCO_3_, 5 mm KH_2_PO_4_, 30 mm D-glucose, 0.7 mm CaCl_2_, 0.4 mm MgSO_4_, and 100 mm D-mannitol (all from Sigma-Aldrich, RRID:SCR_008988), equilibrated by bubbling with 95% O_2_/5% CO_2_ at room temperature. The final Fluo-8, AM concentration was 20 μm, and slice cultures were submerged in bubbled loading solution for 30–60 min before being transferred to a recording chamber, and left for 30 min to wash excess l-aCSF before recoding commenced.

AAV constructs containing genes for GECIs driven by the synapsin promotor (Dana et al., 2019) were obtained from Addgene, and included: pGP-AAV-syn-jGCaMP7s-WPRE (AAV9, Addgene, catalog #104487-AAV9, RRID:Addgene_104487), and pGP-AAV-syn-jGCaMP7f-WPRE (AAV retrograde, Addgene, catalog #104488, RRID:Addgene_104488). The viruses were diluted in ultrapure water and cultures were transduced the day after they were prepared, either by adding a drop of virus solution directly on top of a slice culture (namely, jGCaMP7s drop-transduced, 3.3 × 10^12^ vg/ml) or by injection of ∼50-nl virus solution in the ventrolateral area (jGCaMP7f-retrograde, 2.3 × 10^12^ vg/ml) using a single-barrel glass micropipette. Cultures were used 14–35 d after transduction.

### Electrophysiology

Glass micropipettes were pulled from filamented capillary glass (O.D. 1.5 mm, I.D. 0.86 mm, Harvard Apparatus) using a PUL-100 micropipette puller (World Precision Instruments, RRID:SCR_008593) to a tip resistance of 4–6 MΩ. Patch pipettes were filled with a solution containing the following: 130 mm HCH_3_SO_3_, 130 mm KOH, 10 mm HEPES, 0.4 mm NaGTP, 4 mm Na_2_ATP, 5 mm Na_2_-phosphocreatine, 4 mm MgCl_2_ (all from Sigma-Aldrich, RRID:SCR_008988), 0.01 mm Alexa Fluor 568 hydrazide (ThermoFisher Scientific, RRID:SCR_008452), and 0.5% biocytin (Sigma-Aldrich, catalog #B4261, RRID:SCR_008988). The osmolarity of the patch pipette solution measured 310 mOsm with pH 7.3. Patch pipettes were visually guided to target neurons under visual control using MPC-200 micromanipulator system (Sutter Instruments) on a fixed-stage upright microscope (modified Olympus BX51, Olympus Corporation) under 40× magnification (NA = 0.8, WD = 3.3 mm). Somatic whole-cell patch-clamp recordings were performed in current clamp using an AxoClamp 2B amplifier (Molecular Devices), and the data were digitally acquired at a sampling rate of 5 kHz, with a low-pass filter of 2 kHz. Trains of stimulation pulses (10-ms duration) were given at current levels just high enough to illicit a single AP per pulse.

Field stimulation was performed with a sharp bipolar tungsten electrode, +/− poles spaced ∼400 μm apart, placed on the surface of the culture on one side encompassing a rhythmic preBötC. Trains of unipolar pulses (2-ms duration, 4- to 9-V constant voltage) were used to stimulate fibers and somas between the two poles, using a stimulus isolation unit gated by a waveform generator (A.M.P.I. ISO-Flex isolator, RRID:SCR_018945, AMPI Master 8 generator, RRID:SCR_018889).

Microiontophoresis was performed using a Neurophore-BH-2 instrument (Medical Systems). Filamented theta capillary glass (OD/ID 1.5/1.02 mm, septum 0.2 mm, World Precision Instruments) was used to apply GABA (200 mm, pH 4.35). Pulses were delivered at 0.1 Hz (50- to 500-ms pulse duration) with ejection current: +100 nA, and holding current: −15 nA.

Rhythmic activity in cultures was recorded in high-excitability aCSF (high-aCSF) containing the following: 124 mm NaCl, 3 mm KCl, 5 mm KH_2_PO_4_, 25 mm NaHCO_3_, 25 mm D-glucose, 1 mm ascorbic acid, 1 mm MgCl_2_, and 1.5 mm CaCl_2_ (all from Sigma-Aldrich, RRID:SCR_008988), with pH 7.4, equilibrated by bubbling with 95% O_2_/5% CO_2_. The final concentration of K^+^ was [K^+^]_o_ = 8 mm, which elevates baseline membrane potentials and increases the frequency of spontaneous respiratory rhythm.

In some experiments, the spontaneous rhythm was blocked, while maintaining synaptic transmission, by exchanging high-aCSF with low-excitability aCSF (low-aCSF), which contained the following: 124 mm NaCl, 2 mm KCl, 1.25 mm NaH_2_PO_4_, 25 mm NaHCO_3_, 25 mm D-glucose, 1 mm ascorbic acid, 6 mm MgCl_2_, and 3 mm CaCl_2_, with pH 7.4, equilibrated by bubbling with 95% O_2_/5% CO_2_. In some experiments glutamatergic transmission was also blocked by adding 20 μm NBQX disodium salt (AMPA antagonist, Tocris Bioscience, catalog #1044, RRID:SCR_003689) and 20 μm CPP (NMDA antagonist, Tocris Bioscience, catalog #0173, RRID:SCR_003689) to a superfusing low-aCSF. GABAergic ligands used were muscimol (Sigma-Aldrich, catalog #M1523, RRID:SCR_008988), (+)-bicuculline (Sigma-Aldrich, catalog #14340, RRID:SCR_008988), and (+/−)-Baclofen (Tocris Bioscience) dissolved in water at 10, 20, and 10 mm stock concentration, respectively. Experimental preparations were exposed to GABAergic ligands through addition to the superfusing aCSF or microiontophoretic application.

### Ca^2+^ imaging

Fluorescent Ca^2+^ activity was recorded in wide-field on a fixed-stage upright microscope (modified Olympus BX51), illuminated by a metal halide light source (PhotoFluor II, 89North) or a blue (470 nm) LED light source (M470L2, Thorlabs). Red and green channel fluorescence was visualized using a dual-bandpass filter set (Chroma 59022: excitation dual bandpass 450–490 nm/555–590 nm, emission dual bandpass 500–543 nm/603–665 nm). Red and green channels (for Alexa Fluor 568 hydrazide; Fluo-8, AM; jGCaMP7f/s) were separated during acquisition by manually exchanging an additional excitation filter in the light path (Semrock FF01: bandpass 565–605 nm; Semrock FF02: bandpass 457–487 nm). Time series acquisition was performed with a sCMOS camera (Neo DC-152Q, Andor Technology) controlled by SOLIS software (Andor Technology). Imaging protocols employed 10× (NA 0.3), 20× (NA 0.5), and 40× (NA 0.8) water immersion objectives.

### Biocytin labeling, morphologic reconstruction, and expansion microscopy

Slice cultures containing biocytin injected neurons were drop fixed overnight in 4% paraformaldehyde at 4°C, washed in PBS, labeled with streptavidin-Alexa Fluor 594 (ThermoFisher Scientific, catalog #S11227, RRID:SCR_008452) and cleared in ScaleSQ(5) clearing solution for 3 h at 35°C. Image stacks of labeled neurons, taken at different focal depths, were acquired on an Olympus BX51 microscope using 20× and 40× objectives, a sCMOS camera, a metal halide light source (PhotoFluor II), and a dual-bandpass filter set (Chroma 59022: excitation dual bandpass 450–490/555–590 nm, emission dual bandpass 500–543/603–665 nm). Following extended depth of field transformation of the stacks in ImageJ, to produce a single in-focus image, neuronal morphology was reconstructed by hand, drawing the soma and processes. Some biocytin injected, streptavidin-Alexa Fluor 594-labeled neurons, were processed using a modified protocol for expansion microscopy (Chozinski et al., 2016). In short, the cultures were incubated in 25 mm methacrylic acid N-hydroxysuccinimide ester (MA-NHS; Sigma-Aldrich, catalog #730300, RRID:SCR_008988) for 1 h at room temp., washed in PBS, and incubated in monomer solution containing 8.6% sodium acrylate (Sigma-Aldrich, catalog #408220, RRID:SCR_008988), 2.5% acrylamide (Bio-Rad Laboratories, catalog #1610140, RRID:SCR_008426), 0.15% N,N'-methylenebisacrylamide (BIS, Bio-Rad Laboratories, catalog #1610142, RRID:SCR_008426), and 11.7% NaCl, in PBS for 45 min at 4°C. the cultures were moved to a gelling chamber, and 200 μl gelling solution, consisting of 4 μl 4-hydroxy-TEMPO (4-HT; Sigma-Aldrich, catalog #176141, RRID:SCR_008988), 4 μl tetramethylethylenediamine (TEMED; ThermoFisher Scientific, catalog #17 919, RRID:SCR_008452), 4 μl ammonium persulfate (APS; ThermoFisher Scientific, catalog #17 874, RRID:SCR_008452), and 188 μl monomer solution, was added, and allowed to gel over 2 h at 35°C. The gel surrounding the culture was removed, and the cultures were placed in a digestion buffer containing 1× TAE buffer, 0.5% Triton X-100, 0.8 m guanidine HCl, 50 mm Tris, 1 mm EDTA (all from Sigma-Aldrich), and 13 μl/ml Proteinase K (600 U/ml, ThermoFisher Scientific, catalog #EO0491, RRID:SCR_008452), overnight at 37°C. The sample was then expanded by washing with ultrapure water three times and imaged using the same system as biocytin-injected/streptavidin-Alexa Fluor 594-labeled neurons.

### Experimental design and statistical analysis

Time series of field stimulus-evoked or AP-evoked Ca^2+^ transients were acquired in epochs of 20–50 frames at 10 frames/s, evoked every 15 s. Synaptic Ca^2+^ transients were calculated as the percent change in fluorescence relative to baseline values (ΔF/F_0_). However, it is important to note that fluorescence from a whole-field imaged synapse, in AAV-GECI transduced cultures, is a combination of fluorescence from the synapse, fluorescence from labeled neuronal structures in the foreground and background, and reflected fluorescence from nearby neuronal structures. To correct for these added sources of fluorescence, the background was subtracted frame-by-frame, taken as the equal-sized mean background fluorescence immediately adjacent to the synaptic region of interest (ROI). Since the background fluorescence could be higher or lower than synapse fluorescence in any given experiment, because of GECI labeling of nearby neuronal structures, the starting level of the background signal was set at the starting level of the synapse fluorescence before subtraction. In essence, this background correction algorithm subtracts the curve of nearby fluorescence from the curve of fluorescence over a synapse. Synaptic ROIs were drawn by hand as rectangles of 5 × 5 μm for 20× magnification and 3 × 3 μm for 40× magnification. The mean fluorescence over three initial frames in the synaptic ROI, before stimulus, was defined as F_0_. For each condition, 10–20 sweeps were acquired and averaged before calculation of ΔF/F_0_. Spontaneous rhythmic Ca^2+^ transients over the preBötC were acquired at 5–10 frames/s, and ΔF/F_0_ was calculated by setting F_0_ as the minimal fluorescent value over the time series (5–20 s). Optical data were analyzed offline using ImageJ 1.53d (ImageJ, RRID:SCR_003070), and Igor Pro 8 (IGOR Pro, RRID:SCR_000325). Electrophysiological data were acquired using pClamp 10.3 (pClamp, RRID:SCR_011323) and subsequently analyzed using custom scripts written in Igor Pro 8 (IGOR Pro, RRID:SCR_000325).

All statistical tests were performed using GraphPad Prism 8 (RRID:SCR_002798). Data were verified for normal distribution with Kolmogorov–Smirnov test, and then analyzed using one-way ANOVA with Tukey’s *post hoc* test, unpaired or paired Student’s *t* test as appropriate. Results are reported as mean ± SEM, and as *N* = number of cultures, and *n* = number of presynaptic terminals. Statistical significance was set at *p* < 0.05 based on the culture number for system and neuronal activity data.

## Results

### Improved subcellular Ca^2+^ imaging using GECIs

**Figure 1.**
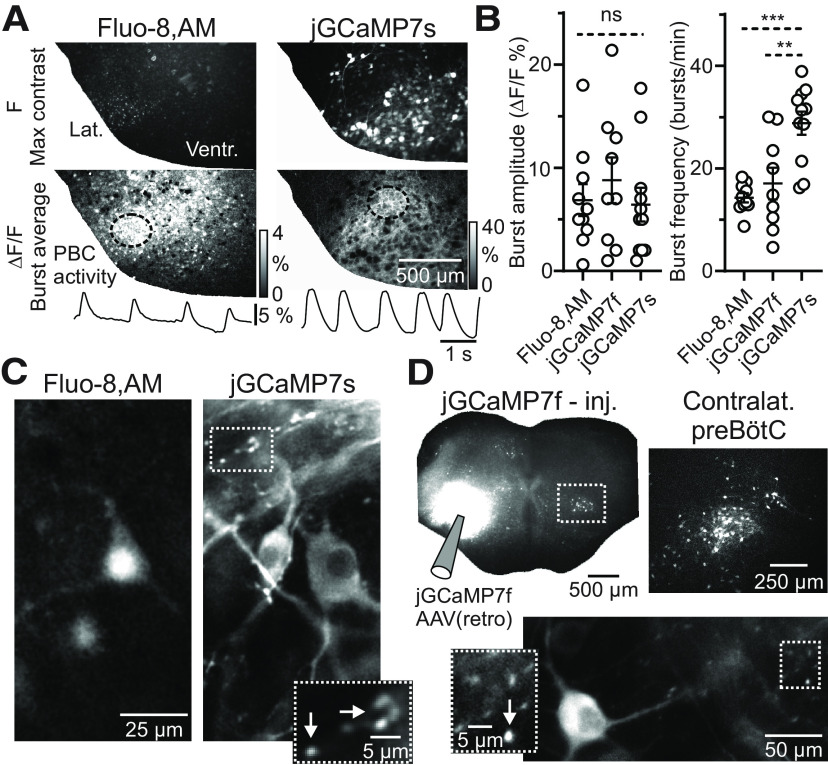
Improved subcellular Ca^2+^ imaging using GECIs in organotypic slice cultures. ***A***, Comparison of Fluo-8, AM-labeled and jGCaMP7s-labeled slice cultures imaged under similar conditions. Left panel, Raw fluorescence [F] from a Fluo-8, AM-labeled culture (top, max contrast), average of cycle-triggered preBötC bursts in the same culture (*N* = 4 bursts, middle), trace of ΔF/F from a ROI over the preBötC (bottom). Right panel, Raw fluorescence [F] from a jGCaMP7s drop-transduced culture (top, max contrast), average of cycle-triggered preBötC bursts in the same culture (*n* = 5 bursts, middle), trace of ΔF/F from a ROI over the preBötC (bottom). Lat., lateral culture border; Ventr., ventral culture border. ***B***, Group data (mean ± SEM) showing similar rhythmic burst amplitudes between Fluo-8, AM-labeled (*N* = 10), jGCaMP7f-labeled (*N* = 9), and jGCaMP7s-labeled (*N* = 11) cultures, but an increased rhythmic burst frequency in jGCaMP7s-labeled cultures. One-way ANOVA, Tukey’s *post hoc* analysis; **p <* *0.01, ***p <* *0.001. ***C***, High-magnification (40× objective) imaging, showing average of cycle-triggered preBötC bursts of Fluo-8, AM-labeled and jGCaMP7s-labeled cultures (max contrast). Note that soma and proximal dendrites are visible in the Fluo-8, AM-labeled culture, but soma, proximal/distal dendritic, and punctate synapse-like structures (arrows in dotted line inset) are visible in the jGCaMP7s-labeled culture. ***D***, top left panel, Low-magnification of entire culture unilaterally injected with AAV (retro)-jGCaMP7f in the ventrolateral culture, showing contralateral labeling of neuron somas in the preBötC area. Lower right, High-magnification (40× objective) imaging, showing average of cycle-triggered preBötC bursts (max contrast) taken from the contralateral preBötC area showing soma, proximal/distal dendritic, and punctate synapse-like labeling (arrow in dotted line inset).

Organotypic slice cultures, containing the preBötC, display bilateral spontaneous synchronized rhythmic bursts of ensemble neuronal activity, which can be visualized by organic fluorescent Ca^2+^ indicators (Phillips et al., 2016) or GECIs (Fig. 1). Here, we studied the potential advantages of two of the most promising sensors of the GCaMP variants (Dana et al., 2019). When comparing Fluo-8, AM with jGCaMP7s drop-transduced and jGCaMP7f preBötC-injected cultures, differences in several rhythmic burst and morphologic parameters were noted (Fig. 1). Imaged under identical conditions, we observed approximately twice of the averaged burst frequency measured over a rhythmic preBötC in jGCaMP7s drop-transduced (28.8 ± 2.2 bursts/min, *N* = 11) cultures compared with both, Fluo-8, AM (14.2 ± 0.9 bursts/min, *N* = 10, one-way ANOVA, *F*_(2,27)_ = 12.84, Tukey’s *post hoc* analysis, *p *=* *0.0002; Fig. 1*B*, right; Movie 1) and jGCaMP7f (17.0 ± 3.1 bursts/min, *N* = 9, Tukey’s *post hoc* analysis, *p *=* *0.0024; Fig. 1*B*, right) preBötC-injected cultures. On the other hand, ΔF/F burst amplitude was similar among these groups (Fluo-8, AM: 6.9 ± 1.5% ΔF/F, jGCaMP7f: 8.8 ± 2.2% and jGCaMP7s: 6.4 ± 1.6%, one-way ANOVA, *F*_(2,27)_ = 0.47, *p *=* *0.62; Fig. 1*B*, left).

Movie 1. Ca^2+^ imaging of spontaneous rhythmic burst activity in the preBötC in organotypic cultures labeled with Fluo-8, AM (left image stack) or jGCaMP7f (right image stack). Note that punctate synapse-like structures display synchronous Ca^2+^ transients in the jGCaMP7f-labeled culture, but only somas and proximal dendrites are visible in the Fluo-8, AM-labeled culture. 10.1523/ENEURO.0154-21.2021.video.1

Higher magnification imaging (40× objective) showed that jGCaMP7s drop-transduced cultures expressed synchronized Ca^2+^ transients in the soma, proximal and distal dendritic regions of neurons in the preBötC, whereas Fluo-8, AM-labeled cultures only showed soma and proximal dendritic labeling (Fig. 1*C*). Surprisingly, synchronized Ca^2+^ transients in punctate synapse-like structures were seen in the top ∼100-μm layer in some cultures intermingled between somas and dendritic profiles (Fig. 1*C*, bottom inset). Sparse-labeling of commissural preBötC neurons was obtained by direct unilateral injection of an AAV-retrograde serotype [AAV(retro)-JGCaMP7f] into the ventrolateral area containing the preBötC (jGCaMP7f preBötC injected; Fig. 1*D*). This resulted in rhythmic neurons in the contralateral preBötC area, with clear punctate synapse-like structures showing synchronized Ca^2+^ transients in phase with the somatic profiles (Fig. 1*D*, bottom inset). From these results, we conclude that using GECIs such as jGCaMP7s and jGCaMP7f, not only allows sensitive tracking of Ca^2+^ activity in neuronal cell bodies and dendrites, but also facilitates measurement of Ca^2+^ dynamics in individual punctate synapse-like structures.

### Axonal arborizations and Ca^2+^ imaging from presumed presynaptic terminals

**Figure 2.**
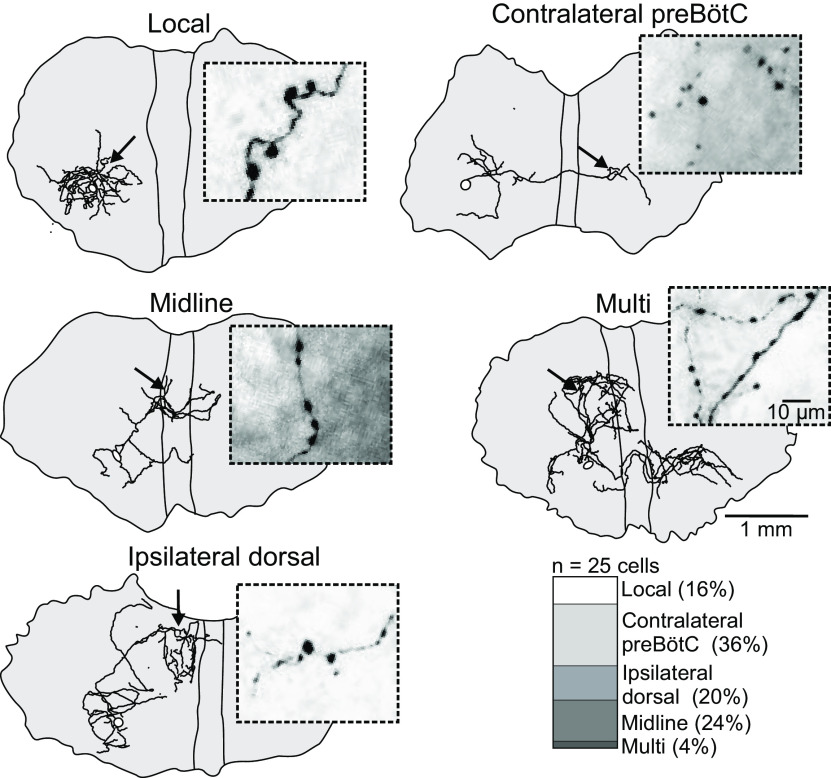
Visualization of axonal arborizations and presumed synaptic boutons in inspiratory preBötC neurons. Manually reconstructed axonal aborizations (and soma: empty circle) of a sample of biocytin-injected rhythmically active preBötC neurons, with projections locally, to the contralateral preBötC, to the midline, to the ipsilateral dorsal area, and multiple projections. Insets, High magnification of presumed synaptic boutons (color inverted, max contrast) in areas indicated with a black arrow in the slice culture. Lower right, Relative projection pattern in 25 biocytin-labeled neurons.

**Figure 3.**
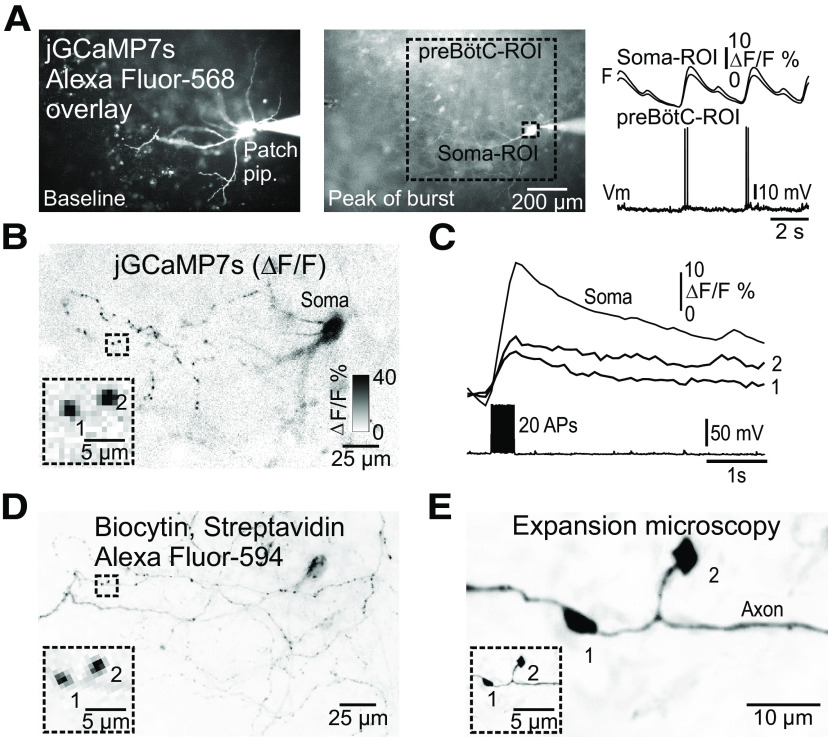
Ca^2+^ imaging from presumed presynaptic terminals in an inspiratory neuron. ***A***, left panel, Live imaging of a rhythmically active preBötC neuron filled with Alexa Fluor 568 through the patch pipette (Patch pip.), overlaid with rhythmic Ca^2+^ transients (jGCaMP7s) in the surrounding preBötC (captured at baseline, and at the peak of a burst). Right panel, Traces of the Vm in the neuron during two cycles of burst activity with spikes on top of each burst, in synchrony with Ca^2+^ transients from the somatic region of the neuron (top trace) and a ROI over the preBötC (lower trace). ***B***, Average of AP-evoked Ca^2+^ transients (20 AP, color inverted, max contrast). Inset, Synchronized Ca^2+^ transients in two presumed presynaptic terminals (1, 2) visible in the neuropil. ***C***, Traces of the AP-evoked Ca^2+^ transients in ROIs over the soma and the two presumed presynaptic terminals (1, 2). Lower trace shows 20 AP. ***D***, Morphology of the same neuron as in ***A***, ***B*** revealed after biocytin labeling with streptavidin Alexa Fluor 594 detection (color inverted, max contrast), re-finding the same two presumed presynaptic terminals (inset). ***E***, Expansion microscopy (expansion factor: 3.5) of the same neuron as in ***A–D***, showing the same two, now expanded, presumed presynaptic terminals. The inset is scaled to the same size as insets in ***B***, ***D***. Note that the two presumed synaptic presynaptic terminals have the appearance of one en passant (1) and one terminal synaptic bouton (2), and that the axon is readily visible.

Considering that preBötC neurons have extensive projections to different brainstem regions implicated in the control of breathing (Tan et al., 2010), we next performed whole-cell patch-clamp recordings with biocytin-filled pipettes from rhythmically active preBötC neurons to visualize axonal projection patterns within the plane of single organotypic slice cultures. In a sample of 25 labeled neurons, 16% showed ipsilateral local projections in the surrounding preBötC, 36% projected to the contralateral preBötC region crossing the midline in the ventral third of the slice culture, 20% projected to the ipsilateral dorsal regions, 24% projected to the midline, and a single neuron (4%) had projections to multiple of the above-mentioned areas (Fig. 2). Importantly, high-magnification imaging of the terminal projection regions showed synapse-like structures associated with axons in all neurons analyzed (Fig. 2, insets). Rhythmically active preBötC neurons showed trains of spikes riding on top of summating synaptic potentials in synchrony with Ca^2+^ transients from the soma and surrounding preBötC (Fig. 3*A*). To corroborate the identity of the synapse-like structures in rhythmically active preBötC neurons labeled with GECIs, the rhythm was stopped by exchanging high-aCSF to low-aCSF. Neurons were then activated by trains of brief depolarizing current pulses (10 ms) evoking 20 APs. This resulted in Ca^2+^ transients in somatodendritic compartments and in discrete synapse-like structures near and far from the neuron (*N* = 4; Fig. 3*B*,*C*). Moreover, the same presynaptic terminals could be identified after processing for biocytin using streptavidin-Alexa Fluor 594, and importantly following further processing using expansion microscopy on the same neurons (*N* = 4 reconstructed and expanded neurons; Fig. 3*D*). The expanded neurons displayed morphologic characteristics of bona fide synaptic boutons of the en passant and terminal type (Fig. 3*E*). When correcting for an expansion factor of 3.5, the maximal diameter of the synaptic boutons was 1.5 ± 0.6 μm (*n* = 83, *N* = 4 neurons). Together, these data suggest that jGCaMP7 sensors may be a useful tool to detect Ca^2+^ activity at the level of individual presynaptic terminals with high reliability.

### AP-dependent and stimulus-dependent changes in presynaptic Ca^2+^ transients

**Figure 4.**
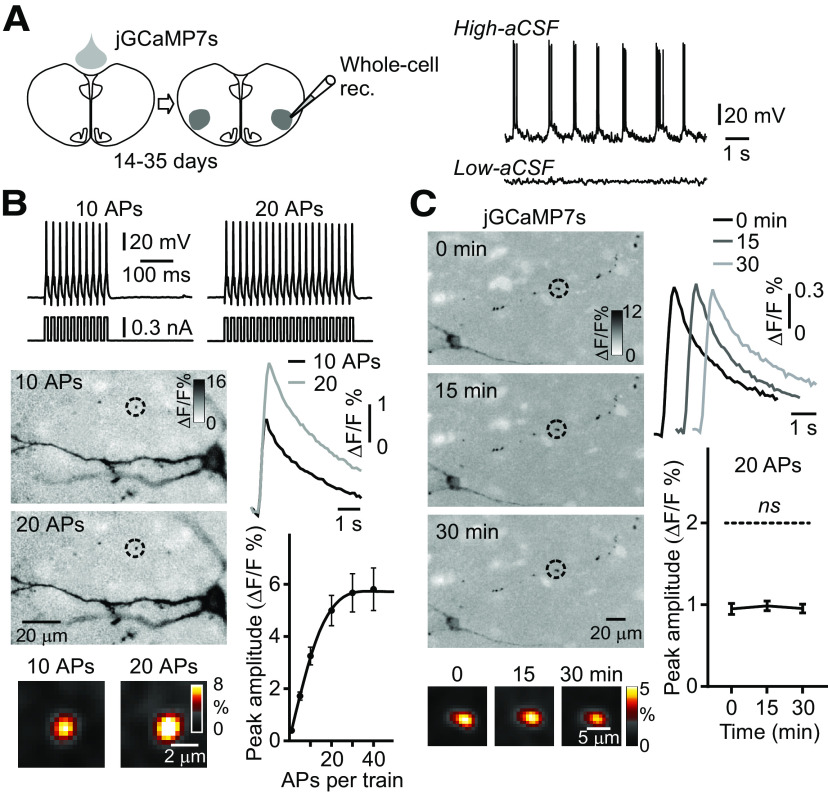
AP-dependent Ca^2+^ transients and Ca^2+^ signal stability in presynaptic terminals in whole-cell recorded preBötC neurons. ***A***, top, Diagram of the experimental paradigm, with drop-transducing jGCaMP7s over the entire culture, followed by whole-cell patch-clamp recording (Whole-cell rec.) from rhythmically active preBötC neurons after 14–35 d. Rhythmic activity in the preBötC was identified in high-aCSF, followed by whole-cell patch-clamp recordings evoking APs with short pulse trains in low-aCSF, where the spontaneous rhythm ceases. ***B***, top recordings, Voltage responses to 10 and 20 depolarizing current steps in preBötC neurons, previously rhythmically active in high-aCSF. Left images, Averaged Ca^2+^ transients in soma and presynaptic terminals, belonging to the neuron in focus, at low and high (inset) magnification during 10 and 20 APs (stimulus-triggered average, color inverted, smart filter, identical contrast settings). Right diagrams, Averaged presynaptic Ca^2+^ transients with 10 and 20 APs (*n* = 30 presynaptic terminals, *N* = 3 cultures). The graph shows the number of APs versus the peak amplitude of the Ca^2+^ transient in single presynaptic terminals (*n* = 30 presynaptic terminals, *N* = 3 cultures, line is a spline fit). ***C***, Stability of the Ca^2+^ signal in presynaptic terminals over 0, 15, 30 min (*n* = 113 presynaptic terminals, *N* = 4 cultures) in a jGCaMP7s drop-transduced culture, and whole-cell recording with 20 current-induced APs (left images) and group data (right diagrams). Note that the Ca^2+^ transient amplitude does not change significantly over 30 min in this experimental paradigm.

To characterize presynaptic Ca^2+^ transients in rhythmically active preBötC neurons two labeling paradigms were used. First, entire slice cultures were jGCaMP7s drop-transduced. After 14–35 d, simultaneous whole-cell patch-clamp and Ca^2+^ imaging were performed on rhythmically active preBötC neurons (Fig. 4*A*, top diagram). Network rhythmic activity was reduced by hyperpolarizing neuronal membrane potentials and increasing the threshold for Na^+^ channel activation with low-aCSF (Phillips et al., 2018). APs were then evoked by trains of brief intracellular current pulses (10-ms duration) with an increasing number of pulses (1–60 with a set time interval of 10 ms; Fig. 4; Movie 2). This protocol resulted in a near-linear rise in the amplitude of presynaptic Ca^2+^ transients (single AP gave ∼0.4% ΔF/F), reaching a saturation maximum above 30 pulses (*n* = 30, *N* = 3 cultures; Fig. 4*B*).

Movie 2. Ca^2+^ transients in a whole-cell patch-clamped preBötC neuron receiving increasing number of depolarizing current steps evoking APs. Left image stack, Soma, dendrites, and presynaptic terminals (color: gray scale at max contrast) at low magnification with concatenated stacks for 1, 5, 10, 20, 30, 40 current pulse steps. Right image stack, Zoomed region (white square in left image) depicting the Ca^2+^ transients (color: smart filter set at max contrast at 40 pulse response) in a few presynaptic terminals and dendrite throughout the pulse train experiment. The video corresponds to the cell shown in Figure 4*B*. 10.1523/ENEURO.0154-21.2021.video.2

**Figure 5.**
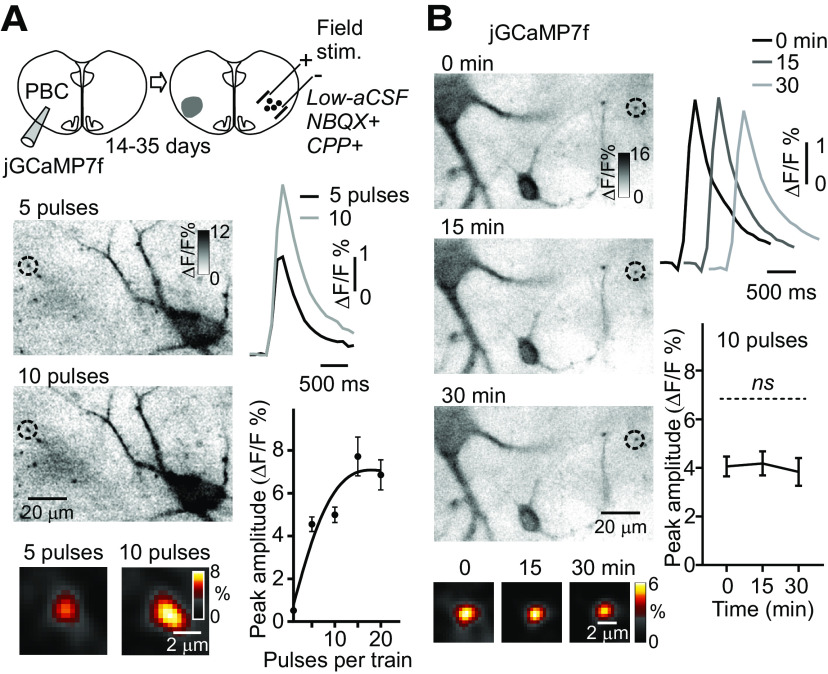
Stimulus-dependent Ca^2+^ transients and Ca^2+^ signal stability in presynaptic terminals in field stimulated preBötC neurons. ***A***, top, Diagram of the experimental paradigm, with a unilateral injection of jGCaMP7f in the ventrolateral area containing the preBötC (PBC), followed by field stimulation in the contralateral preBötC area (Field. stim.) after 14–35 d. The contralateral preBötC region with rhythmic activity was identified in high-aCSF, followed by field stimulation in low-aCSF plus NBQX (20 μm) and CPP (20 μm) to inhibit glutamate-driven group activity. Left images, Averaged Ca^2+^ transients in soma and presynaptic terminals at low and high magnification (insets) in response to field stimulation with 5- and 10-pulse trains (stimulus-triggered average, color inverted, smart filter, identical contrast settings). Right diagrams, Averaged presynaptic Ca^2+^ transients with 5- and 10-pulse trains (*n* = 60 presynaptic terminals, *N* = 6 cultures). The graph shows number of pulses in the pulse train versus peak amplitude of the Ca^2+^ transient in single presynaptic terminals (*n* = 60 presynaptic terminals, *N* = 6 cultures, line is a spline fit). ***B***, Stability of the Ca^2+^ signal in presynaptic terminals over 0, 15, and 30 min (*n* = 39, 39, and 29, respectively, *N* = 4 cultures) in a culture with a unilateral injection of jGCaMP7f, and contralateral preBötC field stimulation with 10 pulses (left images) and group data (right diagrams). Note that the Ca^2+^ transient amplitude does not change significantly over 30 min in this experimental paradigm.

In the second labeling paradigm, cultures were jGCaMP7f unilateral-injected in the ventrolateral preBötC area (Fig. 5*A*). Under high-aCSF conditions, spontaneous synchronized Ca^2+^ transients were observed in presynaptic terminals and somas of commissural neurons in the preBötC, with a larger signal to background separation than in the jGCaMP7s drop-transduced cultures (data not shown). This allowed for a more detailed kinetic analysis of spontaneous Ca^2+^ transients in presynaptic and somatic compartments. Presynaptic Ca^2+^ transients displayed longer 10–90% rise time (417 ± 15 ms, unpaired Student’s *t* test, *t*_(344)_ = 3.70, *p *<* *0.0002), and shorter half-width (629 ± 13 ms, *t*_(344)_ = 9.20, *p* < 0.0001) and 90–10% decay-tau (487 ± 22 ms, *t*_(344)_ = 10.70, *p* < 0.0001, *n* = 243, *N* = 6) compared with somatic Ca^2+^ transients (rise time 325 ± 16 ms; half-width 875 ± 26 ms; decay-tau: 1103 ± 71 ms, *n* = 103, *N* = 6). Rhythmic activity was then reduced by changing the superfusing solution from high-aCSF to low-aCSF, and glutamate-driven group activity was further suppressed by adding NBQX (20 μm), and CPP (20 μm). A bipolar electrode was placed encompassing the contralateral preBötC, with the electrodes placed ∼400 μm apart (Fig. 5*A*, top diagram). Field stimulation was applied in the contralateral preBötC with trains of current pulses (2-ms duration) with a constant amplitude (4–9 V) but an increasing number of pulses (1–30 within a set time frame of 200 ms). This protocol resulted in a near-linear rise in the amplitude of presynaptic Ca^2+^ transients, in commissural neurons captured between the two stimulation electrodes, at an increasing number of pulses, reaching a saturating maximum above 15 pulses (*n* = 60, *N* = 6; Fig. 5*A*).

To determine whether it was possible to use the non-ratiometric GECIs to quantify pharmacologically-induced changes in the amplitude of presynaptic Ca^2+^ transients, the stability of the presynaptic Ca^2+^ signal was determined at 0-, 15-, and 30-min time mark in the two labeling paradigms. Both AP-evoked (20 AP, 0 min: 0.95 ± 0.07% ΔF/F; 15 min: 0.98 ± 0.05%; 30 min: 0.95 ± 0.06%, one-way ANOVA, *F*_(2,9)_ = 0.01, *p *=* *0.99; Fig. 4*C*) and field stimulus-evoked (10 pulses, 0 min: 4.1 ± 0.4% ΔF/F; 15 min: 4.2 ± 0.5%; 30 min: 3.8 ± 0.6%, one-way ANOVA, *F*_(2,8)_ = 0.04, *p *=* *0.95; Fig. 5*B*) Ca^2+^ transients remained essentially unchanged over the three measured time points.

### Effects of ligands of GABA receptors on rhythmic bursting and on Ca^2+^ transients in preBötC presynaptic terminals

**Figure 6.**
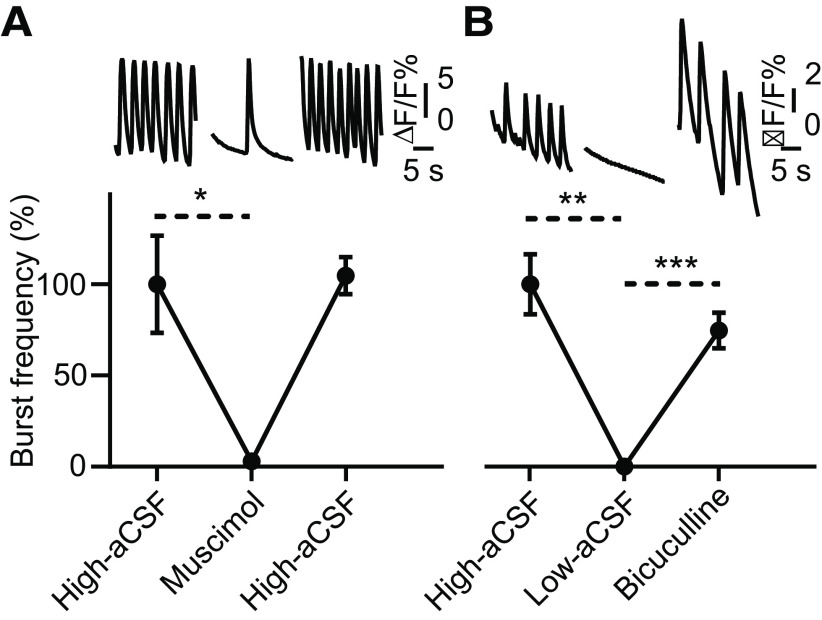
GABA_A_ agonist and antagonist effects on rhythmic bursting in organotypic cultures containing the preBötC. ***A***, top panel, Traces of ΔF/F obtained from the preBötC before, during, and after muscimol (5 μm) application, all in high-aCSF. Bottom graph, Group data (mean ± SEM) showing fully or partially suppressed spontaneous rhythmic burst frequency (in %) during bath application of muscimol in jGCaMP7s-labeled cultures (*N* = 5 cultures). ***B***, top panel, Traces of ΔF/F obtained from the preBötC in high-aCSF, low-aCSF, and after bicuculline (10 μm, in low-aCSF) application. Bottom graph, Group data (mean ± SEM) showing reactivation of the rhythmic burst frequency (in %) during bath application of bicuculline in jGCaMP7s-labeled cultures (*N* = 7 cultures). One-way ANOVA, Tukey’s *post hoc* analysis; *p <* *0.05, **p <* *0.01, ***p <* *0.001.

Previous studies have suggested that inhibitory synaptic transmission affects respiratory rhythm generation and coordination of the inspiratory-expiratory pattern (Janczewski et al., 2013; Marchenko et al., 2016). To determine the role of GABAergic inhibition, in our *in vitro* culture model encompassing the preBötC, jGCaMP7s drop-transduced organotypic slice cultures were bath exposed to the GABA_A_ receptor agonist muscimol (5 μm) or to the antagonist bicuculline (10 μm; Fig. 6). In all cultures exposed to muscimol under high-aCSF conditions, the spontaneous rhythmic activity was fully or partially suppressed (one-way ANOVA, *F* = 12.76, *p *=* *0.017, Tukey’s *post hoc* analysis, *p *=* *0.023, *N* = 5; Fig. 6*A*), but returned when muscimol was washed away. On the other hand, in all cultures treated with bicuculline under low-aCSF conditions, the network rhythmic activity was completely reactivated (one-way ANOVA, *F* = 29.69, *p *=* *0.0004, Tukey’s *post hoc* analysis, *p *=* *0.0004, *N* = 7; Fig. 6*B*).

**Figure 7.**
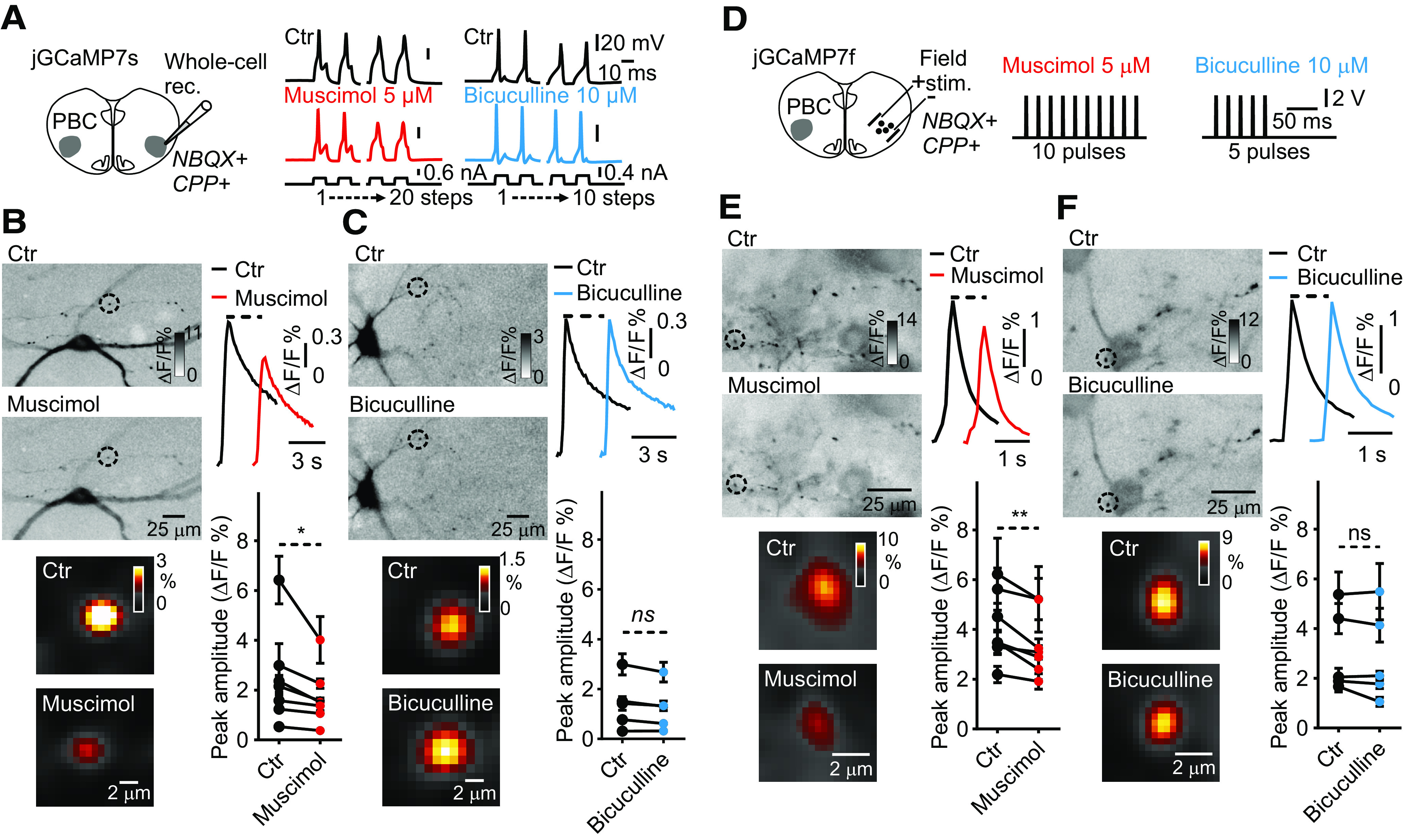
Effects of muscimol and bicuculline on Ca^2+^ transients in preBötC presynaptic terminals. ***A***, ***D***, Diagram of the experimental paradigm. Organotypic cultures containing the preBötC were (***A***) jGCaMP7s drop-transduced or (***D***) jGCaMP7f preBötC injected. After 14–35 d, rhythmic activity in the preBötC region was identified in high-aCSF. Then, under low-aCSF plus NBQX (20 μm) and CPP (20 μm) to inhibit glutamate-driven group activity, (***A***) whole-cell patch-clamp recording (Whole-cell rec.) or (***D***) field stimulation was performed in the contralateral preBötC area (Field. stim.) from rhythmically active neurons in presence or absence of muscimol (red traces) or bicuculline (blue traces). ***B***, ***C***, left images, Averaged Ca^2+^ transients in presynaptic terminals, belonging to the neuron in focus, at low and high (insets) magnification before and after (***B***) muscimol (20 APs) or (***C***) bicuculline (10 APs) application (stimulus-triggered average, color inverted, smart filter, identical contrast settings). Right upper, Averaged presynaptic Ca^2+^ transients with 10 and 20 APs. Right bottom, Summary scatter graphs show (***B***) muscimol and (***C***) bicuculline effects on peak amplitudes of the presynaptic Ca^2+^ transients. ***B***, *n* = 108 presynaptic terminals, *N* = 7 cultures. ***C***, *n* = 50 presynaptic terminals, *N* = 5 cultures. ***E***, ***F***, left images, Averaged Ca^2+^ transients in presynaptic terminals at low and high (insets) magnification in response to field stimulation in presence or absence of (***E***) muscimol or (***F***) bicuculline (10- and 5-pulse trains, respectively, stimulus-triggered average, color inverted, smart filter, identical contrast settings). Right upper, Averaged presynaptic Ca^2+^ transients with 5- and 10-pulse trains. Right bottom, Summary scatter graphs show peak amplitudes of presynaptic Ca^2+^ transients before and after (***E***) muscimol and (***F***) bicuculline application. ***E***, *n* = 69 presynaptic terminals, *N* = 7 cultures. ***F***, *n* = 49 presynaptic terminals, *N* = 5 cultures. Paired Student’s *t* test; *p <* *0.05, **p <* *0.01.

Next, we investigated the effect of muscimol and bicuculline on presynaptic Ca^2+^ transients in jGCaMP7s drop-transduced and jGCaMP7f preBötC-injected preBötC neurons. With this purpose, simultaneous whole-cell patch-clamp (Fig. 7*A*), or field stimulation (Fig. 7*D*), and Ca^2+^ imaging were conducted on rhythmically active preBötC neurons, before (dark traces) and after bath application of muscimol (red traces) or bicuculline (blue traces) under low-aCSF conditions. Active preBötC neurons were first identified in high-aCSF. Glutamatergic transmission was suppressed by adding NBQX and CPP. No significant differences in presynaptic Ca^2+^ transients were found in bicuculline treated preBötC neurons compared with controls, neither in AP-evoked transients (Control (Ctr), 1.4 ± 0.2% ΔF/F, *n* = 50, *N* = 5; bicuculline, 1.2 ± 0.1% ΔF/F, *n* = 50, *N* = 5, paired Student’s *t* test, *t*_(4)_ = 2.43, *p *=* *0.07; Fig. 7*C*), nor in field-evoked transients (Ctr, 3.0 ± 0.3% ΔF/F, *n* = 49, *N* = 5; bicuculline, 2.9 ± 0.3% ΔF/F, *n* = 49, *N* = 5, *t*_(4)_ = 1.28, *p *=* *0.27; Fig. 7*F*). However, preBötC neurons bath treated with muscimol displayed a ∼25% decrease in presynaptic Ca^2+^ transient amplitudes in both, AP-evoked transients (Ctr, 1.6 ± 0.1% ΔF/F, *n* = 108, *N* = 7; muscimol, 1.2 ± 0.1% ΔF/F, *n* = 108, *N* = 7, paired Student’s *t* test, *t*_(6)_ = 2.64, *p *=* *0.03; Fig. 7*B*) and field-evoked transients (Ctr, 4.0 ± 0.3% ΔF/F, *n* = 69, *N* = 7; muscimol, 3.3 ± 0.2% ΔF/F, *n* = 69, *N* = 7, *t*_(6)_ = 4.42, *p *=* *0.004; Fig. 7*E*) compared with controls.

GABA_B_ receptors are well-established modulators of presynaptic terminals in nearly all areas of the brain, including the preBötC (Bongianni et al., 2010; Ghali, 2019). Thus, the effect of the GABA_B_ agonist baclofen (10 μm) on presynaptic Ca^2+^ transients in jGCaMP7f preBötC-injected preBötC neurons was further explored. No significant differences in field-evoked presynaptic Ca^2+^ were found in baclofen treated preBötC neurons compared with controls (Ctr, 3.5 ± 0.4% ΔF/F, *n* = 28, *N* = 3; baclofen, 3.5 ± 0.3% ΔF/F, *n* = 28, *N* = 3, paired Student’s *t* test, *t*_(27)_ = 0.46, *p *=* *0.65). Thus, a possible involvement of GABA_B_ receptors on the presynaptic Ca^2+^ transients was unlikely to have contributed to the presynaptic inhibition observed here.

**Figure 8.**
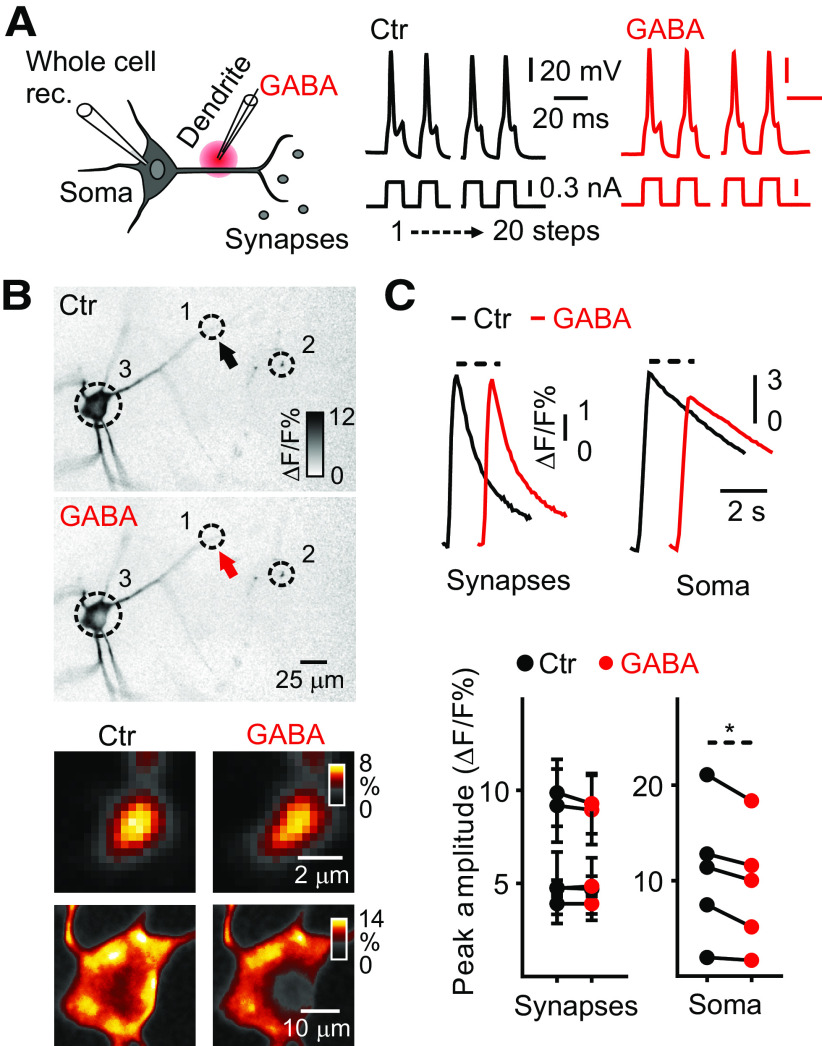
Effects of microiontophoretically applied GABA to dendrites on Ca^2+^ transients in rhythmically active preBötC neurons. ***A***, left, Diagram of the experimental paradigm. Whole-cell patch-clamp recording (Whole-cell rec.) and local microiontophoresis of GABA on dendrites of rhythmic preBötC neurons. Right, Voltage response to depolarizing current steps in preBötC neurons, previously rhythmically active in high-aCSF, before (dark line) and after GABA application (red line). ***B***, Representative images of averaged Ca^2+^ transients in presynaptic terminals and soma, belonging to the neuron in focus, at low and high (insets) magnification during 20 APs (stimulus-triggered average, color inverted, smart filter, identical contrast settings). (1) Dendrites proximal to the GABA-pipette (solid black arrow: no GABA; solid red arrow: GABA application); presynaptic terminals (2) and soma (3) distal to the GABA-pipette. ***C***, upper, Averaged presynaptic and somatic Ca^2+^ transients with 20 Aps. Bottom, Summary scatter graphs show peak amplitudes of synaptic and somatic Ca^2+^ transients before and after GABA application; *n* = 35 presynaptic terminals, 5 somas. Paired Student’s *t* test; *p <* *0.05.

The muscimol-induced reduction in presynaptic Ca^2+^ transients could in principle be because of transmission failure, where evoked somatic APs do not reach synaptic terminals. To test this possibility, we performed simultaneous whole-cell patch-clamp recordings and Ca^2+^ imaging from rhythmically active preBötC neurons, before (dark traces) and after (red traces) local microiontophoresis of GABA applied to dendrites, synapses, and soma. This was performed under low-aCSF, NBQX^+^, and CPP^+^ conditions, using a dual-barrel theta pipette containing GABA (200 mm) in one barrel and extracellular aCSF in the other (Fig. 8). When GABA was locally applied (≤500 μm, for 50–500 ms; Fig. 8*B*, solid red arrow near dotted circle 1) to dendrites, immediately before Ca^2+^ imaging, an ∼15% reduction of the somatic Ca^2+^ transient amplitude (Ctr, 10.9 ± 3.2% ΔF/F, *n* = 5 somas; GABA, 9.3 ± 2.8% ΔF/F, *n* = 5 somas, *t*_(4)_ = 3.69, paired Student’s *t* test, *p *=* *0.02; Fig. 8*B*,*C*) was observed, whereas presynaptic Ca^2+^ transients (Ctr, 6.3 ± 0.6% ΔF/F, *n* = 35, *N* = 5; GABA, 6.2 ± 0.6% ΔF/F, *n* = 35, *N* = 5, *t*_(4)_ = 1.28, *p *=* *0.27; Fig. 8*B*,*C*) of rhythmically active preBötC neurons were not affected.

**Figure 9.**
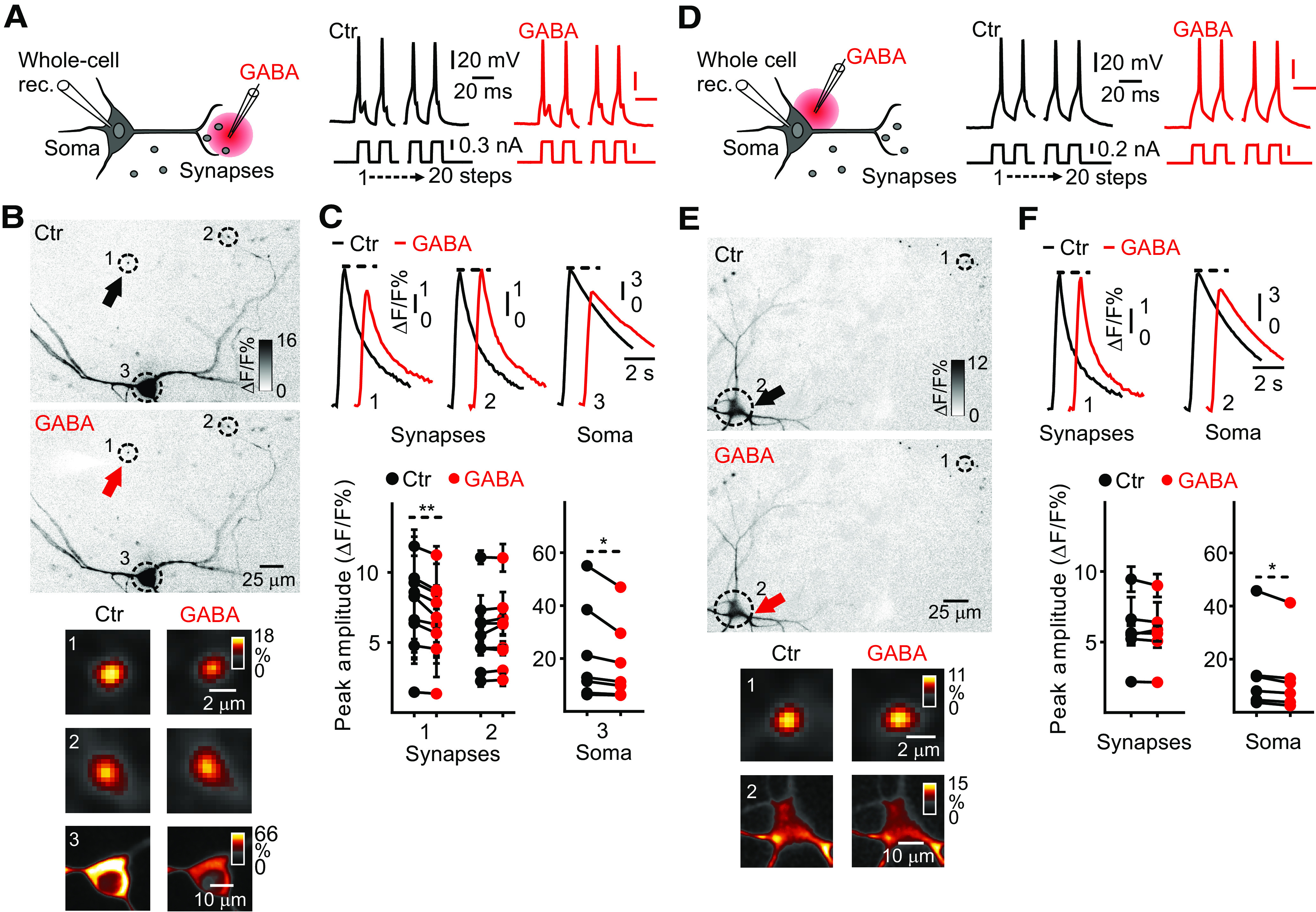
Effects of microiontophoretically applied GABA on presynaptic and somatic Ca^2+^ transients in active preBötC neurons. ***A***, ***D***, left, Diagram of the experimental paradigm. Whole-cell patch-clamp recording (Whole-cell rec.) and local microiontophoresis (≤500 μm) of GABA to subsets of presynaptic terminals (***A***) or to soma (***D***) of rhythmic preBötC neurons. Right, Voltage response to depolarizing current steps in preBötC neurons, previously rhythmically active in high-aCSF, before (dark line) and after GABA application (red line). ***B***, ***E***, Representative images of averaged Ca^2+^ transients in soma and presynaptic terminals, belonging to the neuron in focus, at low and high (insets) magnification during 20 APs (stimulus-triggered average, color inverted, smart filter, identical contrast settings). (1) Presynaptic terminals proximal (***B***) or distal (***E***) to the GABA-pipette (solid black arrow: no GABA; solid red arrow: GABA application); (2) presynaptic terminals distal (***B***) or soma proximal (***E***) to pipette; (3) soma distal (***B***) to the GABA-pipette. ***C***, ***F***, upper, Averaged presynaptic and somatic Ca^2+^ transients with 20 APs. Bottom, Summary scatter graphs show peak amplitudes of synaptic and somatic Ca^2+^ transients before and after GABA application. ***C***, *n* = 27 proximal and *n* = 55 distal presynaptic terminals, 7 somas. ***F***, *n* = 61 distal presynaptic terminals, 6 somas. Paired Student’s *t* test; *p <* *0.05, **p <* *0.01.

Next, GABA was locally applied to a subset of presynaptic terminals (≤500 μm; Fig. 9*B*, solid red arrow near dotted circle 1), the Ca^2+^ transient amplitude decreased by ∼8% on those synapses compared with controls (Ctr, 7.6 ± 0.9% ΔF/F, *n* = 27, *N* = 9; GABA, 7.0 ± 0.8% ΔF/F, *n* = 27, *N* = 9, *t*_(8)_ = 4.81, *p *=* *0.0013; Fig. 9*B*,*C*). Importantly, no significant changes were observed in the Ca^2+^ transient amplitude of more distally located (>500 μm; Fig. 6*B*, dotted circle 2) presynaptic terminals in the same field of view (Ctr, 5.1 ± 0.3% ΔF/F, *n* = 55, *N* = 9; GABA, 5.3 ± 0.3% ΔF/F, *n* = 55, *N* = 9, *t*_(8)_ = 1.65, *p *=* *0.13; Fig. 9*B*,*C*). Surprisingly, a small but significant decrease in somatic Ca^2+^ transient amplitude was also observed during microiontophoresis of GABA to presynaptic terminals (dotted circle 3, Ctr, 21.7 ± 6.9% ΔF/F, *n* = 7, *N* = 7; GABA, 18.3 ± 5.6% ΔF/F, *n* = 7 somas, *t*_(6)_ = 2.54, *p *=* *0.04; Fig. 9*B*,*C*). Finally, local somatic application (≤500 μm; Fig. 9*E*, solid red arrow near dotted circle 2) of GABA decreased ∼12% the somatic Ca^2+^ transient amplitude compared with controls (Ctr, 14.9 ± 6.4% ΔF/F, *n* = 6 somas; GABA, 13.1 ± 5.8% ΔF/F, *n* = 6 somas, *t*_(5)_ = 3.01, *p *=* *0.02; Fig. 9*E*,*F*). On the other side, no significant changes were observed in Ca^2+^ transient amplitude of distally located (Fig. 9*E*, dotted circle 1) presynaptic terminals when GABA was applied somatically (Ctr, 5.6 ± 0.3% ΔF/F, *n* = 61, *N* = 6; GABA, 5.5 ± 0.3% ΔF/F, *n* = 61, *N* = 6, *t*_(5)_ = 0.87, *p *=* *0.42; Fig. 9*E*,*F*).

## Discussion

Recent improvements in GECIs allow accurate measurements of Ca^2+^ in neuronal cell bodies and dendrites with high spatial and temporal resolution. The present study demonstrates that it is possible to target novel GECIs, such as jGCaMP7s and jGCaMP7f, to individual presynaptic terminals in inspiratory neurons in organotypic slice cultures containing the preBötC with high reliability. This novel approach, combined with whole-cell patch-clamp and field stimulation, provides new evidence that GABAergic presynaptic inhibition exists in the neurons comprising the breathing oscillator. This evidence includes reduced presynaptic Ca^2+^ transients in inspiratory neurons and commissural neurons projecting to the contralateral preBötC, in response to bath applied muscimol and microiontophoresis of GABA under non-rhythmic conditions. These observations indicate a role of GABAergic presynaptic inhibition in rhythmic breathing activity.

The use of small-molecule fluorescent Ca^2+^ indicator dyes (Tsien, 1981; Tsien et al., 1982) revolutionized neuronal Ca^2+^ imaging since these indicators are easily loaded into the intracellular space by membrane-permeant ester (acetoxymethyl, AM) forms (Tsien, 1981; Tsien et al., 1982; Grynkiewicz et al., 1985), or through a pipette containing cell-impermeant salt forms (Tank et al., 1988; Müller and Connor, 1991). Loading distal subcellular compartments, such as presynaptic terminals is difficult but has been achieved in some systems using dextran-conjugated indicators (Brenowitz and Regehr, 2012). However, recent advances in the design of GECIs may provide a solution to the challenge of recording Ca^2+^ transients in small subcellular compartments. Thus, earlier variants of GECIs (GCaMP6 fused to synaptophysin) can report on presynaptic Ca^2+^ in neurons in primary culture (Brockhaus et al., 2019; Ferron et al., 2020), and in the calyx of Held synapse following viral injection of GCaMP6 variants three weeks prior (Singh et al., 2018). Biolistic transfection, electroporation, or AAV transduction of hippocampal, hypothalamic, and cortical organotypic slice cultures with plasmids encoding GCaMP3/6 variants or RFP-based GECIs, result in strong somatodendritic labeling, but no apparent presynaptic labeling (Podor et al., 2015; Shen et al., 2018; Gasterstädt et al., 2020; Kim et al., 2020). Interestingly, jGCaMP7 variants show labeling of presynaptic boutons of the Drosophila larval neuromuscular junction (Dana et al., 2019). This prompted us to combine these latest jGCaMP7 variants with AAV-based expression in organotypic slice cultures to enable direct visualization of presynaptic Ca^2+^ in preBötC neurons. Indeed, we find that transduction with AAV9 and AAV-retro encoding jGCaMP7s and jGCaMP7f, respectively, driven by the Syn promoter, results in strong labeling of soma, dendrites, and notably in synapse-like structures that were identified as synaptic boutons of the en passant and terminal type using expansion microscopy. Morphologic reconstruction of biocytin-labeled preBötC neurons in our brainstem cultures shows that the projection pattern of these neurons, and synaptic terminal fields, match that of *in vivo*-labeled neurons at the rostrocaudal level of the preBötC (Yang and Feldman, 2018). Interestingly, jGCaMP7s drop-transduced cultures showed a faster rhythm than Fluo-8, AM or/and jGCaMP7f preBötC-injected cultures, but we hypothesize that this may result from deleterious effect of the loading methods on the preBötC network, as AM loading required lipophilic compounds and preBötC-injections may affect the integrity of the ipsilateral network. However, this methodological advance allows for experiments that can address control of presynaptic Ca^2+^ in neurons comprising the central pattern generator (CPG) network, and has several advantages in the current embodiment, but also some disadvantages. Here, using conventional wide-field illumination microscopy, labeled synapses were only readily visible in the top ∼100-μm layer of the cultures, because of diffraction of light coming from deeper structures. However, we surmise that the use of confocal and multi-photon imaging may resolve presynaptic terminals located deeper. Transducing organotypic cultures from Cre transgenic animals with AAVs encoding for Cre-dependent GECI variants may allow for visualization of presynaptic Ca^2+^ in genetically specified neurons. The GECIs used here are non-ratiometric Ca^2+^ indicators, which represents a disadvantage, since bleaching, concentration differences, and optical path problems make quantification difficult. Careful control experiments and corrective calculations allowed for comparisons of drug effects performed over ∼15-min experimental windows, but this time window was also constrained by small movements of the tissue. Time-lapse imaging (data not shown) reveals mobile cells, presumed to be microglia, push their way through the cultures, which combined with small flow-dependent movement of the membrane-attached cultures, and slight thermal movement of the optical system induced a drifting focus in some experiment. This could be corrected for, if it was in the *x-y*-plane, but invalidated quantification if it was in the *z*-plane.

Breathing movements are very complex, and need to change constantly during, e.g., exercise, coughing, swallowing, and vocalizing, fine-tuning respiratory frequency and depth. These state-dependent changes require inhibitory neural mechanisms that can temporarily stop or modulate the activity of premotor neurons driving respiratory motor pools. However, the role of synaptic inhibition in the breathing CPG is controversial, since pharmacological experiments, applying GABAergic and glycinergic antagonists to the VRC in rodents, have given conflicting results (Paton and Richter, 1995; Shao and Feldman, 1997; Pierrefiche et al., 1998; Bongianni et al., 2010; Janczewski et al., 2013; Marchenko et al., 2016). Some studies show a critical role of synaptic inhibition in rhythm generation (Schmid et al., 1996; Pierrefiche et al., 1998; Marchenko et al., 2016), whereas others show that rhythm can persist following blockade of synaptic inhibition in the preBötC and BötC (Janczewski et al., 2013). Nonetheless, the available data shows that synaptic inhibition plays a role in shaping the temporal sequence and amplitude of the neural outputs from the respiratory controller to motor pools driving respiratory muscles. An important insight into the role of synaptic inhibition in the preBötC comes from optogenetic experiments selectively activating inhibitory preBötC neurons (Vgat+ putative GABAergic and glycinergic neurons), demonstrating that phasic inhibition is critical for maintaining a normal rapid preBötC rhythm (Cregg et al., 2017; Baertsch et al., 2018). The mechanism that mediates this effect may involve curtailing the refractory period following inspiratory bursts, allowing rebound excitation to activate subsequent bursts. Activity of GABAergic neurons also appears to regulate synchronization of rhythmogenic preBötC neurons by changing the conductance state of the neurons (Ashhad and Feldman, 2020). GABAergic and glycinergic ionotropic receptor mechanisms localized at the somatodendritic membranes might mediate these effects by hyperpolarizing and shunting the membrane, and thereby reduce the effect of excitatory input and affect the balance of inhibition and excitation in the entire network of glutamatergic, GABAergic, and glycinergic neurons. Under low-excitability conditions, GABA_A_ receptor blockade can restart spontaneous preBötC rhythm in acutely prepared brainstem slices (Ashhad and Feldman, 2020). Here, we find that muscimol blocks rhythm, and bicuculline, in low-aCSF, can restart rhythm in organotypic cultures containing the preBötC, suggesting indeed that GABAergic inhibition might play a role in rhythmogenesis. However, when excitability was lowered with low-aCSF, and glutamatergic synaptic transmission was blocked with NBQX and CPP, there was no effect of GABA_A_ receptor blockade on presynaptic Ca^2+^ transients. This implies that baseline release of GABA under these conditions is too low to induce presynaptic inhibition, and that the GABAergic presynaptic inhibition requires a rhythmic network, or neurons with heightened excitability. When GABA was applied locally to presynaptic terminals, a small decrease was also noted in the somatic Ca^2+^ transient amplitude. This is surprising, and could result from diffusion of GABA to nearby dendrites since separate experiments showed that dendritically applied GABA reduces somatic Ca^2+^ transient amplitudes. Together, these observations suggest that GABA acting on the somatodendritic membrane may shunt, and profoundly change the electrotonic compactness of preBötC neurons, affecting AP-induced Ca^2+^ influx. The GABAergic inhibition of presynaptic Ca^2+^ transients discovered here in inspiratory preBötC neurons, including commissural preBötC neurons, adds to the ways that synaptic inhibition can influence the network activity in the neural kernel of inspiratory rhythm generation. We propose that the GABAergic reduction in presynaptic Ca^2+^ transients leads to a reduced transmitter output from the synaptic terminal, as less Ca^2+^ is available for the release machinery. How activation of presynaptic GABA_A_ receptors couple to a reduced Ca^2+^ influx is unknown from these experiments, but likely result from hyperpolarization and shunting of the presynaptic membrane reducing the depolarizing effect of arriving APs, and thereby reducing the activation of presynaptic voltage-gated Ca^2+^ channels. It is also unknown whether somatodendritic and presynaptic GABAergic inhibition arises from the same presynaptic GABAergic neurons, or whether there might be more focused projection patterns relying on subsets of presynaptic and subsets of somatodendritic projecting neurons. This series of experiments did not determine whether the neurons that show presynaptic inhibition where themselves glutamatergic, GABAergic, or glycinergic. Thus, an open question is still whether GABAergic presynaptic inhibition targets a subset of these classes of neurons. An important caveat of this series of experiments is that the GABAergic presynaptic inhibition was observed under non-rhythmic conditions, and it remains to be determined whether it occurs cyclically under normal breathing rhythm, and thus contribute to the synaptic dynamics of the breathing CPG.

In summary, we demonstrate that jGCaMP7 variants label presynaptic terminals in organotypic slice cultures of the brainstem, and that the breathing CPG includes neurons that show GABAergic inhibition of presynaptic Ca^2+^ transients. This approach may be used to investigate the dynamics of presynaptic Ca^2+^ in other functioning networks of the CNS.

## Synthesis

Reviewing Editor: Kevin Wickman, University of Minnesota Decisions are customarily a result of the Reviewing Editor and the peer reviewers coming together and discussing their recommendations until a consensus is reached. When revisions are invited, a fact-based synthesis statement explaining their decision and outlining what is needed to prepare a revision will be listed below. The following reviewer(s) agreed to reveal their identity: Naohiro Koshiya, Swen Hülsmann.

This is a resubmission of the paper “GABAergic inhibition of presynaptic Ca2+ transients in respiratory preBötzinger neurons in organotypic slice cultures” While there are notable improvements in this revision, and the technical aspects of the study are impressive, a major reviewer concern was largely unaddressed:

"Interpretation/conclusions: While the authors present a large body of data, presynaptic inhibition is really directly addressed only in Figure 9. And even here, the data show only that the boutons have functional GABAA receptors.”

The Abstract remains unchanged from the original and begins with: “GABAergic somatodendritic inhibition in the preBötzinger Complex...is involved in respiratory rhythmogenesis and patterning.” This is immediately followed by the paper’s central focus: “Nevertheless, whether GABA acts distally on presynaptic terminals, evoking presynaptic inhibition is unknown.” This logical sequencing frames the latter as a counterpart of the former, where GABAergic mechanisms operate in the functional, rhythmic neural network. The Abstract continues: “Here, we address THIS PROBLEM by measuring presynaptic Ca2+ transients...”. The relevant data presented in the manuscript, however, describe effects of externally applied GABAA agonist muscimol and leave THIS PROBLEM (i.e., the existence of ongoing/tonic GABAergic presynaptic inhibition) unaddressed. The Significance Statement restates the central question: “...it is unclear whether central pattern generator networks (CPGs) controlling rhythmic movements make use of modulation of presynaptic Ca2+”.

The authors are advised to revise the manuscript as needed (Abstract, Significance Statement, Results, Discussion) to ensure a better alignment of interpretations/conclusions with the evident limitations of the approaches used in the study. To be clear, the data presented in the study more accurately support the claim that GABAA-dependent presynaptic inhibition of inspiratory neurons in the preBotC may be involved in respiratory rhythmogenesis and patterning. Any claim that GABAA-dependent presynaptic inhibition of inspiratory neurons in the preBotC shapes respiratory rhythmogenesis and patterning should be removed from the manuscript barring the addition of new data that directly addresses this question.

## Author Response

Manuscript: eNeuro eN-NWR-0154-21

Title: GABAergic inhibition of presynaptic Ca2+ transients in respiratory preBötzinger neurons in organotypic slice cultures

Dear Dr. Kevin Wickman,

Please find enclosed the revised version of the manuscript eN-NWR-0154-21. In this revised version, all points raised by the referees were carefully addressed. We thank the reviewers for their comments. None of the additional changes and analysis have modified our original conclusions.

Response to Reviews Comments:

Synthesis of Reviews:

Computational Neuroscience Model Code Accessibility Comments for Author (Required): N/A

Significance Statement Comments for Author (Required):

Modification to soften conclusions are required (see comments below) Comments on the Visual Abstract for Author (Required):

N/A

Synthesis Statement for Author (Required):

This is a resubmission of the paper “GABAergic inhibition of presynaptic Ca2+ transients in respiratory preBötzinger neurons in organotypic slice cultures” While there are notable improvements in this revision, and the technical aspects of the study are impressive, a major reviewer concern was largely unaddressed:

"Interpretation/conclusions: While the authors present a large body of data, presynaptic inhibition is really directly addressed only in Figure 9. And even here, the data show only that the boutons have functional GABAA receptors.”

The Abstract remains unchanged from the original and begins with: “GABAergic somatodendritic inhibition in the preBötzinger Complex...is involved in respiratory rhythmogenesis and patterning.” This is immediately followed by the paper’s central focus: “Nevertheless, whether GABA acts distally on presynaptic terminals, evoking presynaptic inhibition is unknown.” This logical sequencing frames the latter as a counterpart of the former, where GABAergic mechanisms operate in the functional, rhythmic neural network. The Abstract continues: “Here, we address THIS PROBLEM by

measuring presynaptic Ca2+ transients...”. The relevant data presented in the manuscript, however, describe effects of externally applied GABAA agonist muscimol and leave THIS PROBLEM (i.e., the existence of ongoing/tonic GABAergic presynaptic inhibition) unaddressed. The Significance Statement restates the central question: “...it is unclear whether central pattern generator networks (CPGs) controlling rhythmic movements make use of modulation of presynaptic Ca2+”.

The authors are advised to revise the manuscript as needed (Abstract, Significance Statement, Results, Discussion) to ensure a better alignment of interpretations/conclusions with the evident limitations of the approaches used in the study. To be clear, the data presented in the study more accurately support the claim that GABAA-dependent presynaptic inhibition of inspiratory neurons in the preBotC may be involved in respiratory rhythmogenesis and patterning. Any claim that GABAA-dependent presynaptic inhibition of inspiratory neurons in the preBotC shapes respiratory rhythmogenesis and patterning should be removed from the manuscript barring the addition of new data that directly addresses this question.

Answer: We fully agree with the reviewer that the question of whether GABAergic presynaptic inhibition operates during cyclic breathing activity in the preBötzinger remains unanswered in this study. We have made changes in the relevant paragraphs in the abstract (p1, l5,6,14,17), significance statement (p2, l29,31), introduction (p4, l76), and discussion (p19,l411,414, 445-446,515-519) to make this point clear. In particular, it is now explicitly stated in the discussion, that an important caveat of this series of experiments is that the GABAergic presynaptic inhibition was observed under non- rhythmic conditions, and it remains to be determined whether it occurs cyclically under normal breathing rhythm, and thus contribute to the synaptic dynamics of the breathing CPG.
